# Using Phenotypic, Biochemical, and Molecular Methods to Identify *Candida* Species Isolated in a Tertiary Hospital in Mexico

**DOI:** 10.3390/epidemiologia7040112

**Published:** 2026-08-17

**Authors:** Karen del Carmen Morales-Ramírez, María Guadalupe Frías-De-León, Esperanza Duarte-Escalante, Teresa Soledad Cid-Pérez, Manuel Huerta-Lara, Raúl Avila-Sosa, Ricardo Munguía-Pérez

**Affiliations:** 1 Posgrado en Ciencias Ambientales, Instituto de Ciencias, Benemérita Universidad Autónoma de Puebla, Puebla 72000, Mexico; karen_morales10@hotmail.com (K.d.C.M.-R.); manuel.huerta@correo.buap.mx (M.H.-L.); 2 Laboratorio de Micología Molecular, Unidad de Investigación Biomédica, Hospital Regional de Alta Especialidad de Ixtapaluca (HRAEI), Servicios de Salud del Instituto Mexicano de Seguro Social para el Bienestar (IMSS-BIENESTAR), Carretera Federal México-Puebla Km 34.5, Ixtapaluca 56530, Mexico; magpefrias@gmail.com; 3 Departamento de Microbiología y Parasitología, Facultad de Medicina, Universidad Nacional Autónoma de México, México City 04510, Mexico; dupe@unam.mx; 4 Facultad de Ciencias Químicas, Benemérita Universidad Autónoma de Puebla; Puebla 72000, Mexico; teresa.cid@correo.buap.mx; 5 Instituto de Ciencias, Benemérita Universidad Autónoma de Puebla, Puebla 72000, Mexico

**Keywords:** candidiasis, non-*albicans Candida*, hospital epidemiology, immunocompromised patients

## Abstract

Background/Objectives: *Candida* species are among the leading causes of fungal infections, especially in immunosuppressed patients. In recent years, the increase in non-*albicans* species has altered the epidemiological landscape, requiring updated local surveillance data. Therefore, this study aims to analyze the epidemiological pattern of *Candida* spp. involved in various clinical manifestations. Methods: This was a retrospective observational descriptive study of clinical *Candida* isolates recovered from patients at a single tertiary care hospital in Puebla, Mexico, between 2021 and 2022. Sociodemographic and clinical data were collected from medical records. *Candida* spp. isolates were recovered from the hospital’s clinical specimen collection and identified using conventional methods and phylogenetic analysis. Results: Ninety isolates were recovered from a total of 88 patients during the study period. The majority of patients were women (60.2%), and among the age groups, the largest proportion corresponded to older adults (≥60 years, 25%). *Candida* was most frequently isolated from the urinary tract (47.7%), followed by the respiratory tract (19.3%) and the vulvovaginal tract (14.7%). *Candida albicans* was the predominant species (51.1%), followed by *Nakaseomyces glabratus* (32.2%) and other species such as *Pichia kudriavzevii* (5.5%), *Candida tropicalis* (3.3%), *Candida dubliniensis* (3.3%), *Candida parapsilosis* (2.2%), and *Candida orthopsilosis* (1.1%). The study showed discrepancies between conventional identification methods and molecular analysis. Of the 33 isolates evaluated using culture methods, only 21 matched the identification by PCR and phylogenetic analysis. In the case of the BD Phoenix automated system, 20 matches with and 13 discrepancies from the molecular tests were recorded. Conclusions: Our findings revealed the significant presence of species belonging to the non-*albicans* family in specific clinical contexts, highlighting the urgent need to monitor the epidemiological profile. The observed discrepancies between phenotypic and molecular identification methods suggest that species identification based solely on phenotypic approaches should be interpreted with caution.

## 1. Introduction

Candidiasis is an infection caused by several species of the genus *Candida*, a group of yeasts that are normally part of the human microbiome; however, this infection can also be caused by other species such as *P. kudriavzevii* and *N. glabratus*. However, under certain conditions, these species can behave as opportunistic fungal pathogens, responsible for infections of the skin, mucous membranes, deep tissues, and internal organs [1,2]. Among the most common clinical manifestations are superficial cutaneous forms, such as intertriginous infections and diaper dermatitis [3], as well as mucocutaneous presentations, including oral candidiasis, vaginitis, and balanitis [4]. In contrast, invasive candidiasis, commonly associated with hospital care, refers to bloodstream infections (candidemia) and other deep-seated forms that can affect any organ due to hematogenous dissemination of the yeast [5]. These infections may present as intra-abdominal abscesses, peritonitis, pneumonia, pyelonephritis, endocarditis, or meningitis, particularly in immunocompromised patients [6,7].

The prevalence and distribution of *Candida* species are influenced by multiple factors, including clinical context, geographic location, medication use, the presence of invasive devices, immunosuppressive or chronic diseases, and nutritional status [8,9,10,11]. As a result of these variables, the isolation of non-*albicans* species as causative agents of candidiasis has increased, particularly in patients with underlying vulnerabilities [8].

However, *C. albicans* remains the main species involved in fungal infections, accounting for approximately 70% of all cases of mycosis worldwide [12]; moreover, its involvement in invasive candidiasis in hospital settings is associated with a high mortality rate, close to 40%, even with antifungal treatment [5]. According to studies conducted in various hospitals in Mexico, *C. albicans* remains the most frequent agent in cases of candidiasis; however, non-albicans species constitute a significant proportion of isolates, with *N. glabratus* being the second most common, and *C. parapsilosis* and *P. kudriavzevii* being the most frequent [13,14,15,16]. Urogenital candidiasis is the only notifiable mycosis in Mexico. According to the Ministry of Health morbidity yearbook, 118,089 new cases were reported nationwide in 2021, including 7814 cases in the state of Puebla [17]. Given this burden and the variability according to clinical site and population, as well as the emergence of antifungal resistance, it is essential to maintain continuous epidemiological monitoring and employ combined methods (phenotypic, biochemical, and molecular methods) for accurate species identification, early detection of resistance profiles, and appropriate guidance of prevention and treatment policies.

The World Health Organization (WHO) published a list of priority fungal pathogens, classified into three groups in response to the growing threat of emerging resistance and treatability of infection. *Candida auris* and *C. albicans* are included in the critical group due to their high prevalence and ability to cause serious infections. The high-priority group includes *N. glabratus* (formerly *Candida glabrata*), *C. tropicalis*, and *C. parapsilosis*, while *P. kudriavzevii* (formerly *Candida krusei*) is listed in the medium-priority group [18]. *C. auris* is one of the *Candida* spp. that has shown the most marked increase in incidence in recent years. Coupled with its reduced susceptibility to conventional antifungal agents, it has emerged as a major cause of candidemia associated with high mortality rates [19,20].

These findings underscore the importance of continuous mycological surveillance, particularly in tertiary care hospitals caring for high-risk patient populations. The identification of species and their clinical distribution is essential for improving therapies and designing strategies to control *Candida* species infections. Therefore, this study seeks to analyze the epidemiological pattern of *Candida* spp. involved in various clinical manifestations in patients at a tertiary hospital in Puebla, Mexico.

## 2. Materials and Methods

### 2.1. Population Characteristics

This was a retrospective observational descriptive study of clinical *Candida* isolates recovered from patients at a single tertiary care hospital in Puebla, Mexico, between 2021 and 2022. According to medical records, *Candida* isolates were recovered from clinical samples collected from patients with diverse types of infections.

### 2.2. Inclusion and Exclusion Criteria for Clinical Isolates

Clinical isolates were included if they met the following criteria: (1) samples collected in the period of 2021–2022; (2) microbiological identification of at least one *Candida* species, as well as other species previously classified as *Candida*, in clinical samples; and (3) availability of complete patient data, including age, sex, type of specimen, medical service, and diagnosis. The exclusion criterion was (1) non-viable or contaminated isolates. The documented variables included patient age and sex, type of candidiasis, microbiological identification of *Candida* species, and clinical history (chronic diseases, allergies, and prior surgical procedures). Colonization was distinguished from infection based on clinical context and diagnostic criteria, and mixed cultures were recorded and analyzed separately to avoid misclassification.

### 2.3. Identification of Candida Isolates via Biochemical and Phenotypic Tests

The initial biochemical identification of clinical isolates was performed using CHROMagar™ *Candida* chromogenic medium (BD Difco™) (Becton, Dickinson and Company, Detroit, MI, USA) and Phoenix™ ID (Becton, Dickinson and Company, Sparks, MD, USA) panels for yeasts, processed in the BD Phoenix™ M50 automated system. In the case of presumptive *C. albicans* isolates identified by any of the biochemical methods, additional physiological tests were performed to differentiate them from *C. dubliniensis*. These included the germ tube test in human serum, chlamydoconidia production on cornmeal agar, and thermotolerance testing on SDA at 45 °C with an incubation period of 24 to 48 h [21]. For morphological confirmation of the presumptive isolates, microscopic examinations were carried out using cotton blue staining and wet mount observation. These techniques allowed for the assessment of characteristic structures such as germ tubes, chlamydoconidia, and blastoconidia. Isolates that tested negative in physiological tests were identified as *C. dubliniensis,* with the thermotolerance test being the determining criterion for their classification.

As a quality control measure, reference strains were included, namely, *C. tropicalis* ATCC^®^ 66029™ and *N. glabratus* ATCC^®^ 2001™, to validate the reproducibility and specificity of the tests performed. Identification through the automated BD Phoenix™ system was prioritized for non-*albicans Candida* isolates, as it provides a more detailed and accurate characterization in a clinical context. However, when isolates were identified as *C. albicans* by either method, chromogenic medium or BD Phoenix™, the complementary phenotypic tests were considered determinative.

### 2.4. Genotypic Identification of Candida Species

DNA Extraction from Yeast

All isolates that showed discrepant phenotypic identification results (Table 1 and Appendix A.1) were analyzed using molecular methods. To validate the results obtained, the reference strains *N. glabratus* ATCC^®^ 2001™ and *C. albicans* ATCC^®^ 18804™ were included as controls. Both clinical isolates and reference strains were cultured in 3 mL of YPD medium (1% yeast extract, 2% peptone, 2% dextrose) and incubated at 30 °C for 24 h. Subsequently, the yeasts were harvested by centrifugation at 8000 rpm for 1 min, and the supernatant was discarded. Genomic DNA extraction was performed using a Fungi/Yeast Genomic DNA Isolation Kit (Norgen Biotek Corp., Thorold, ON, Canada), strictly following the manufacturer’s instructions.

Species Identification of Candida via PCR

PCR for the identification of eight *Candida* species (*C. albicans*, *N. glabratus*, *C. tropicalis*, *C. parapsilosis*, *C. krusei*/*P. kudriavzevii*, *Meyerozyma guilliermondii*, *Clavispora lusitaniae*, and *C. dubliniensis*) was performed following the conditions described by García-Salazar et al. [22].

The primers used were CandF (5′-AGCTTGCGTTGATTACGTCCCTGCCC-3′) and CandR (5′-TTCACTCGCCGCTACTAAGGCAATCCC-3′). PCR reactions were carried out in a final volume of 25 µL containing 10 ng of DNA from each isolate, 200 μM dNTPs (Jena Bioscience), 1.5 mM MgCl_2_, 100 pmol of each oligonucleotide (Cand-F and Cand-R) (Sigma-Aldrich, Saint Louis, MO, USA), 1 U of Taq DNA Polymerase (Jena Bioscience, Jena, Germany), and 1X PCR buffer. As positive controls, 10 ng of DNA from the reference strains *C. albicans* ATCC^®^ 18804™ and *N. glabratus* ATCC^®^ 2001™ was used. Deionized water (Milli-Q, Merck KGaA, Danvers, MA, USA) was used as a negative control. The amplification program was as follows: one cycle of 3 min at 94 °C; thirty-three cycles of 30 s at 95 °C, 30 s at 55 °C, and 1 min at 72 °C; and a final extension of 5 min at 72 °C. After amplification, 6 μL of PCR products was analyzed by electrophoresis on a 1.5% agarose gel (Axygen Bioscience, Union City, CA, USA) stained with Midori Green (NIPPON Genetics Tokio, Japan), using 0.5X TBE buffer at 70 V for two hours. A 100 bp DNA Ladder (PROMEGA, Madison, WI, USA) was used as a molecular size marker. Gel images were captured using a gel documentation system (Cleaver Scientific Ltd., Rugby, Warwickshire, UK). Molecular identification of the yeast isolates was based on the specific amplicon size corresponding to each species (Table 1).

PCR Amplification of the ITS Region for Candida Species Identification Through Phylogenetic Analysis

Isolates that showed discrepancies in identification according to physiological, chromogenic, and automated tests were identified through phylogenetic analysis based on sequences obtained from the amplification of the ITS1-5.8S-ITS2 region of the ribosomal DNA, as described in the scientific literature [23,24,25,26]. Universal primers ITS1 (5′-TCCGTAGGTGAACCTGCGG-3′) and ITS4 (5′-TCCTCCGCTTATTGATATGC-3′) (Sigma-Aldrich, Saint Louis, MO, USA) were used under the conditions described by Mirhendi et al. (2006) [27]. PCR reactions were carried out in a final volume of 25 µL containing 10 ng of DNA from each isolate, 200 μM dNTPs (Jena Bioscience, Jena, Germany), 1.5 mM MgCl_2_, 100 pmol of each oligonucleotide, 1 U of Taq DNA Polymerase (Jena Bioscience, Jena, Germany), and 1X PCR buffer. As positive controls, 10 ng of DNA from the reference strains *C. albicans* ATCC^®^ 18804™ and *N. glabratus* ATCC^®^ 2001™ was used. Deionized water (Milli-Q, Merck KGaA, Danvers, MA, USA) served as the negative control. The amplification program was as follows: one cycle of 5 min at 94 °C; twenty-five cycles of 30 s at 94 °C, 45 s at 56 °C, and 1 min at 72 °C; and a final extension of 7 min at 72 °C. Following PCR, 6 μL of the amplification products was analyzed by electrophoresis on 1.5% agarose gel (Axygen Bioscience, Union City, CA, USA), stained with Midori Green (NIPPON Genetics, Tokio, Japan), using 0.5X TBE buffer at 70 V for two hours. A 100 bp DNA Ladder (PROMEGA, Madison, WI, USA) was used as the molecular size marker. Gel images were captured using a gel documentation system (Cleaver Scientific Ltd., Rugby, Warwickshire, UK).

Sequencing of Amplified Fragments

The PCR products obtained from the eleven *Candida* isolates were sent for bidirectional sequencing (Psomagen, Inc., Rockville, MD, USA). Sequence analysis was performed using BioEdit software version 7.2 (https://bioedit.software.informer.com/7.2/, accessed on 23 January 2025), which enabled manual verification of the forward and reverse sequences for each sample and the generation of consensus sequences. Each consensus sequence was analyzed using the Basic Local Alignment Search Tool (BLAST), version 1.4.9 [28] (https://blast.ncbi.nlm.nih.gov/Blast.cgi, accessed on 5 February 2025), to confirm species identity. In addition, the sequence alignments of the ITS region were analyzed considering the percentages of similarity, identity, and expectation to corroborate the homology between the obtained sequences.

Phylogenetic Analysis

Reference ITS sequences from various *Candida* species (see Table 2) were included to construct a phylogenetic tree using both the maximum likelihood (ML) method and MEGA X software (www.megasoftware.net, accessed on 7 October 2025) [29,30]. Branch support was estimated using a bootstrap analysis with one thousand replicates [31]. The sequences obtained with the ITS marker were deposited in GenBank (access numbers: PZ325472, PZ325473, PZ325474, PZ325475, PZ325476, PZ325477, PZ325478, PZ325479, PZ325480, PZ325481, and PZ325482) (Supplementary Table S1).

### 2.5. Data Analysis

The collected data were organized in a database for analysis using descriptive statistics. The frequency of qualitative variables is expressed as percentages. The analysis was performed using Microsoft Excel version 365 and Minitab^®^ Statistical Software version 22. The results are presented in frequency tables, grouped bar charts, and pie charts to facilitate visualization of species distribution and its relationship to different variables.

## 3. Results

### 3.1. Patient Characteristics

The individuals included in this study received care at a tertiary-level hospital located in the city of Puebla. Most were residents of neighborhoods in the northern area of Puebla de Zaragoza municipality, as well as certain regions of the state of Tlaxcala. All were affiliated with Mexico’s public healthcare system, which provides free medical services. A total of 88 patients with yeast isolates belonging to the genus *Candida* were analyzed. In two of them, two different species were identified. The distribution by sex and age group is presented in Table A2 in Appendix A.2. The mean age of the study population was 46.3 years, ranging from 13 to 89 years. Most cases occurred in older adults (≥60 years), followed by the 40–59 age group. The percentage of patients diagnosed with candidiasis was higher among females (53/88; 60%) than males (35/88; 40%).

### 3.2. Sample Type and Clinical Manifestations

#### 3.2.1. Urinary Tract

**Figure 1:**
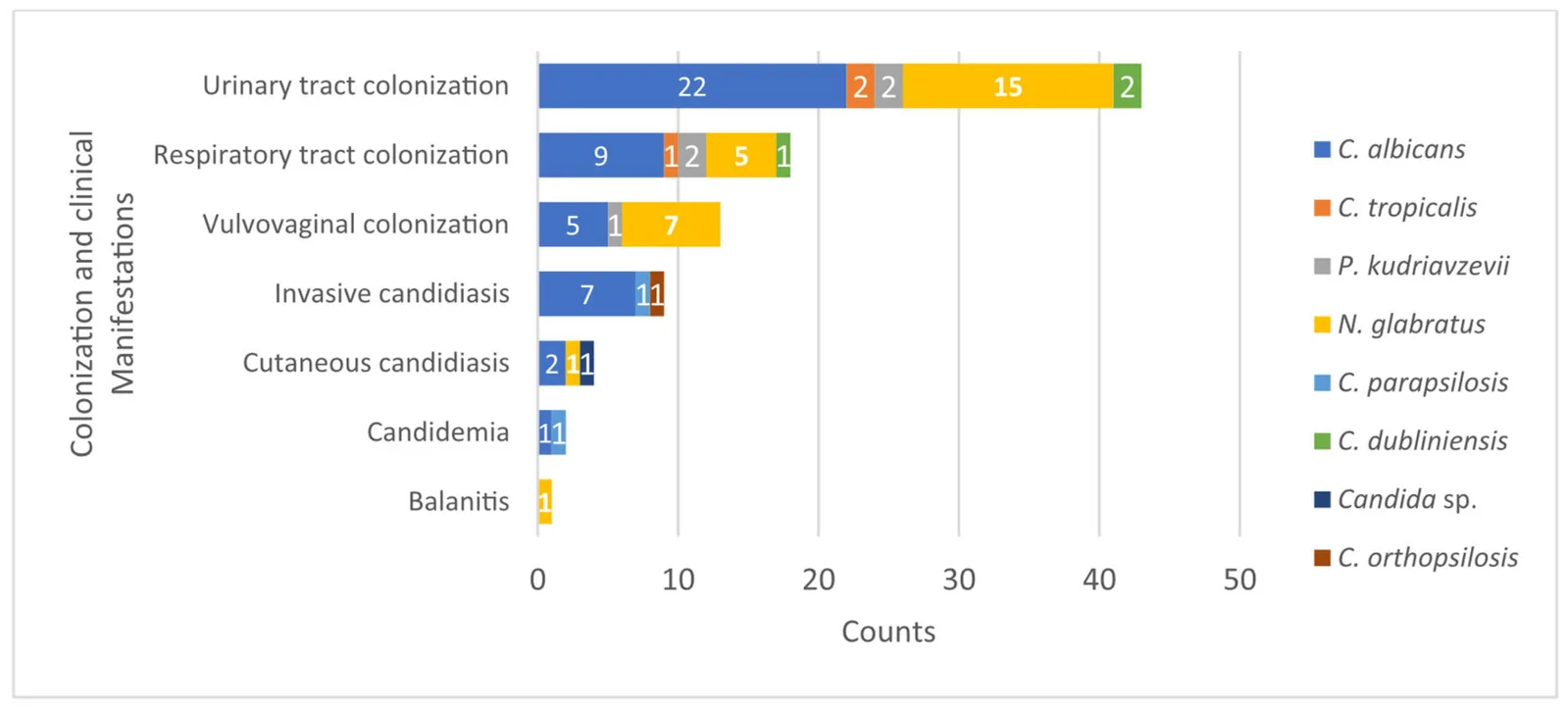
Distribution of *Candida* species isolated in different types of infection and colonization. The frequency of yeast isolates associated with different cases is shown: colonization of the urinary, respiratory, and vaginal tracts; invasive candidiasis; cutaneous candidiasis; candidemia; and balanitis.

*Candida* isolates were most frequently recovered from the urinary tract, accounting for 42 of 88 cases (47.7%), comprising 27 women and 15 men. Five yeast species were identified, with *C. albicans* (n = 22) and *N. glabratus* (n = 15) predominating. Less frequently recovered species included *C. tropicalis* (n = 2), *C. dubliniensis* (n = 2), and *P. kudriavzevii* (n = 2) (Figure 1). One mixed culture contained both *N. glabratus* and *P. kudriavzevii*.

Patients ranged in age from 15 to 89 years, with the highest number of cases occurring in patients aged ≥60 years (n = 15), followed by those aged 49–59 years (n = 10) and 39–49 years (n = 10). Common underlying conditions included chronic kidney disease, diabetes mellitus, pyelonephritis, COVID-19, vascular disease, and hepatobiliary disorders. Most urine specimens (30/42) were collected from patients with indwelling urinary catheters, whereas 12 were obtained without catheterization.

#### 3.2.2. Respiratory Tract

Seventeen *Candida* spp. isolates were recovered from respiratory samples, accounting for 19.3% of the total cases analyzed. These recoveries occurred in seven female and ten male patients. Identified species included *C. albicans* (n = 9), *N. glabratus* (n = 5), *C. tropicalis* (n = 1), *C. dubliniensis* (n = 1), and *P. kudriavzevii* (n = 2) (Figure 1). It should be noted that *N. glabratus* and *P. kudriavzevii* were part of a mixed culture from a male patient diagnosed with pneumonia. Greater involvement was observed in the 39+ age group, with higher frequency in patients aged 60 or over. Clinical history revealed that most patients had underlying diseases. Respiratory diseases were observed in six patients, including pneumonia (n = 2), atypical pneumonia (n = 1), chronic obstructive pulmonary disease (COPD) (n = 1), and SARS-CoV-2 infection (n = 2). Systemic comorbidities included type 2 diabetes mellitus (n = 1), cerebrovascular disease (n = 1), chronic kidney disease (n = 1), liver failure (n = 1), intestinal obstruction (n = 1), and arterial insufficiency (n = 1). One patient was diagnosed with HIV infection, and another had a healthcare-associated infection (HAI). Three patients lacked a primary clinical diagnosis in the reviewed medical records.

#### 3.2.3. Vulvovaginal Candidiasis and Balanitis

Vaginal and urethral samples with yeast-like growth and Gram-stained slides showing yeast, blastoconidia, or pseudohyphae were accepted according to the criteria of Basso et al. [32]. Thirteen cases of vulvovaginal candidiasis were identified (14.7% of the total), with *N. glabratus* being the most prevalent species (n = 7), followed by *C. albicans* (n = 5). Additionally, one isolation of *P. kudriavzevii* was detected (Figure 1). Affected patients ranged in age from 13 to 49 years, predominantly between 19 and 29 years. Relevant clinical histories included one case of diabetic ketoacidosis, ten patients were pregnant at diagnosis, and two had no significant clinical history. Within genital-origin samples, one case of balanitis caused by *N. glabratus* was reported in a 59-year-old male patient without a relevant medical history.

#### 3.2.4. Invasive Candidiasis and Candidemia

A candidemia episode was defined as the isolation of *Candida* spp. from at least one blood culture obtained by peripheral venipuncture. According to the EORTC/MSGERC definitions, a candidemia episode fulfills the criteria for proven invasive fungal disease [33]. Invasive candidiasis was defined as the recovery of *Candida* spp. from cultures of other normally sterile sites, supported by compatible clinical findings, risk factors, and microbiological tests. Nine patients (10.2%) presented with invasive candidiasis: five females and four males, aged between 15 and 63 years. The most affected age group was 29 to 39 years. Relevant clinical conditions included severe intra-abdominal infections: pancreatitis (n = 1), peritonitis associated with peritoneal dialysis (n = 2), peritonitis linked to a Tenckhoff catheter (n = 1), abdominal sepsis (n = 1), and septic shock (n = 2). Two additional cases were severe soft tissue infections: Fournier’s gangrene (n = 1) and Ludwig’s angina (n = 1). Etiological agents included *C. albicans* (n = 7), *C. parapsilosis* (n = 1), and *C. orthopsilosis* (n = 1). Two episodes of candidemia were documented: one was documented in a 13-year-old male patient with atypical pneumonia, in whom *C. albicans* was isolated; the other involved *C. parapsilosis* from a 52-year-old male patient with chronic kidney disease.

#### 3.2.5. Cutaneous Candidiasis

Cultures of wound samples were evaluated using the Q index [34]. Cutaneous candidiasis was identified in four patients (4.5%; 4/88), including three men (aged 30 to 63 years) and one woman (51 years). Infections appeared to be associated with specific sites of barrier disruption or underlying conditions, including postoperative wound dehiscence, nephrostomy, and Penrose drain. One patient had a history of cerebrovascular disease. The species isolated from these sites were *C. albicans* (n = 2), *N. glabratus* (n = 1), and one *Candida* spp. isolate. Figure 1 shows the distribution of *Candida* species according to clinical manifestation type (see Table A2). Overall, *C. albicans* was the predominant species in most clinical presentations.

### 3.3. Identification of Candida Isolates Using Biochemical and Physiological Tests

Yeast isolate identification was conducted using the BD Phoenix™ automated system, CHROMagar™ *Candida* chromogenic medium, and conventional physiological tests (chlamydoconidia production, germ tube formation in human serum, and thermotolerance). These methods allowed for the discrimination of eight *Candida* species, namely, *C. albicans* (n = 48; 53.3%), *N. glabratus* (n = 22; 24.4%), *C. tropicalis* (n = 5; 5.5%), *P. kudriavzevii* (n = 4; 4.4%), *C. parapsilosis* (n = 3; 3.3%), *C. dubliniensis* (n = 5; 5.5%), *C. kefyr* (n = 1; 1.1%), and *C. melibiosica* (n = 1; 1.1%), and one *Candida* sp. (n = 1; 1.1%).

### 3.4. PCR-Based Identification

The PCR method was used selectively on 33 isolates that showed discrepancies between physiological and biochemical tests. Table 3 shows the concordance and discrepancies between the identification methods. PCR *Cand* allowed the direct identification of 22 isolates, of which 15 were identified as *C. albicans*. Among these, two had been characterized as *N. glabratus* by the chromogenic medium, and most showed negative results for chlamydoconidia production. The remaining seven isolates were identified as *N. glabratus*, although four of them had previously been characterized as *P. kudriavzevii* by the chromogenic medium. The remaining 11 isolates that could not be unambiguously resolved by the PCR Cand assay (22 directly identified + 11 unresolved = 33 discrepant isolates) were subsequently characterized by ITS region sequencing and phylogenetic analysis (Section 3.4.1).

**Figure 2:**
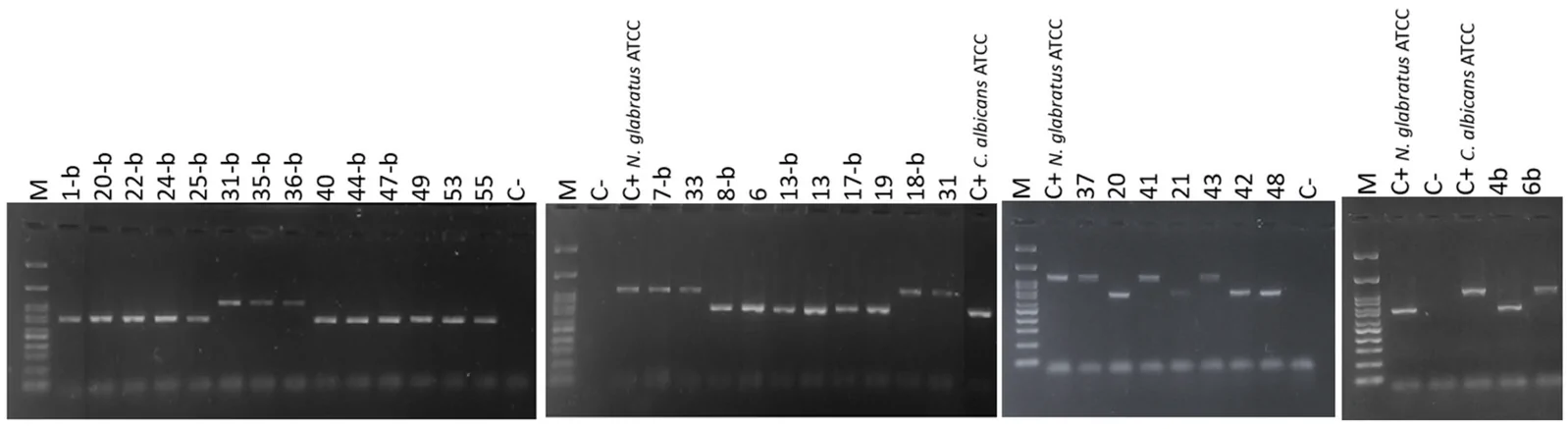
Agarose gel electrophoresis of PCR assay for *Candida* isolates. *Candida albicans*: lanes 6, 13, 19, 20, 21, 42, 48, 49, 53, 4b, 8b, 20b, 22b, 24b, and 47b. *Nakaseomyces glabratus*: lanes 31, 33, 37, 6b, 31b, 35b, and 36b. (C−) Negative control. (M) 100 bp DNA ladder.

Amplification of specific regions showed band patterns consistent with those of the reference strains, which allowed validation of the isolate assignments. Figure 2 shows the bands corresponding to the amplified products of *C. albicans* and *N. glabratus*. A 100 bp molecular size marker (M) and a negative control (C−) were included to verify the specificity of the assay.

#### 3.4.1. PCR Amplification of the ITS Region for Candida Species Identification via Phylogenetic Analysis

Eleven yeast species that showed discrepancies in their identification using physiological, biochemical, and PCR tests were subjected to phylogenetic analysis to confirm their classification. The resulting sequences were deposited in GenBank, and the corresponding accession numbers are available in Supplementary Table S1.

The phylogenetic tree generated by the maximum likelihood method grouped the isolates as follows: Isolates 1b, 7b, and 25b clustered with reference sequences of *C. parapsilosis* (LC389747.1, EU564206, EU552502.1, LC389733.1, KY102294.1, KM014590.1, EU564200.1, and EU552499.1). Isolate 44b grouped with reference sequences of *C. orthopsilosis* (KY102262.1, KC846140.1, KF738147.1, and EU557370.1), with a bootstrap value of 100%. Isolate 17b grouped with *C. tropicalis* reference sequences (KY102470.1, OR859805.1, MH793862.1, and MH545915.1), with a bootstrap value of 100%. Isolates 13b and 40 clustered with *P. kudriavzevii* reference sequences (KC886644.1, MK394162.1, KP674762.1, MK592834.1, MZ677198.1, and FJ231424.1), with a bootstrap support of 100%. Isolates 41, 43, 55, and 18b grouped with *N. glabratus* reference sequences (KC408956.1, MF187263.1, MF187253.1, KP675693.1, MF371417.1, KY575045.1, KC408962.1, KP674942.1, KP675655.1, LC317496.1; KP675517.1; OW988753.1; ON479770.1, and KP068740), with a bootstrap support of 99% (Figure S1).

The eleven discrepant isolates (those with inconsistent identification results from biochemical, physiological, and PCR-based methods) were reclassified via phylogenetic analysis as follows: four isolates were identified as *N. glabratus* (41, 43, 55, and 18b), which BD Phoenix™ had previously misidentified as *C. melibiosica*, *C. kefyr*, *N. glabratus*, and *C. albicans*; two isolates were identified as *P. kudriavzevii* (40, and 13b); three were identified as *C. parapsilosis* (7, 1b, and 25b); one was identified as *C. tropicalis* (17b); and one was identified as *C. orthopsilosis* (44b) (Figure S1).

**Figure 3:**
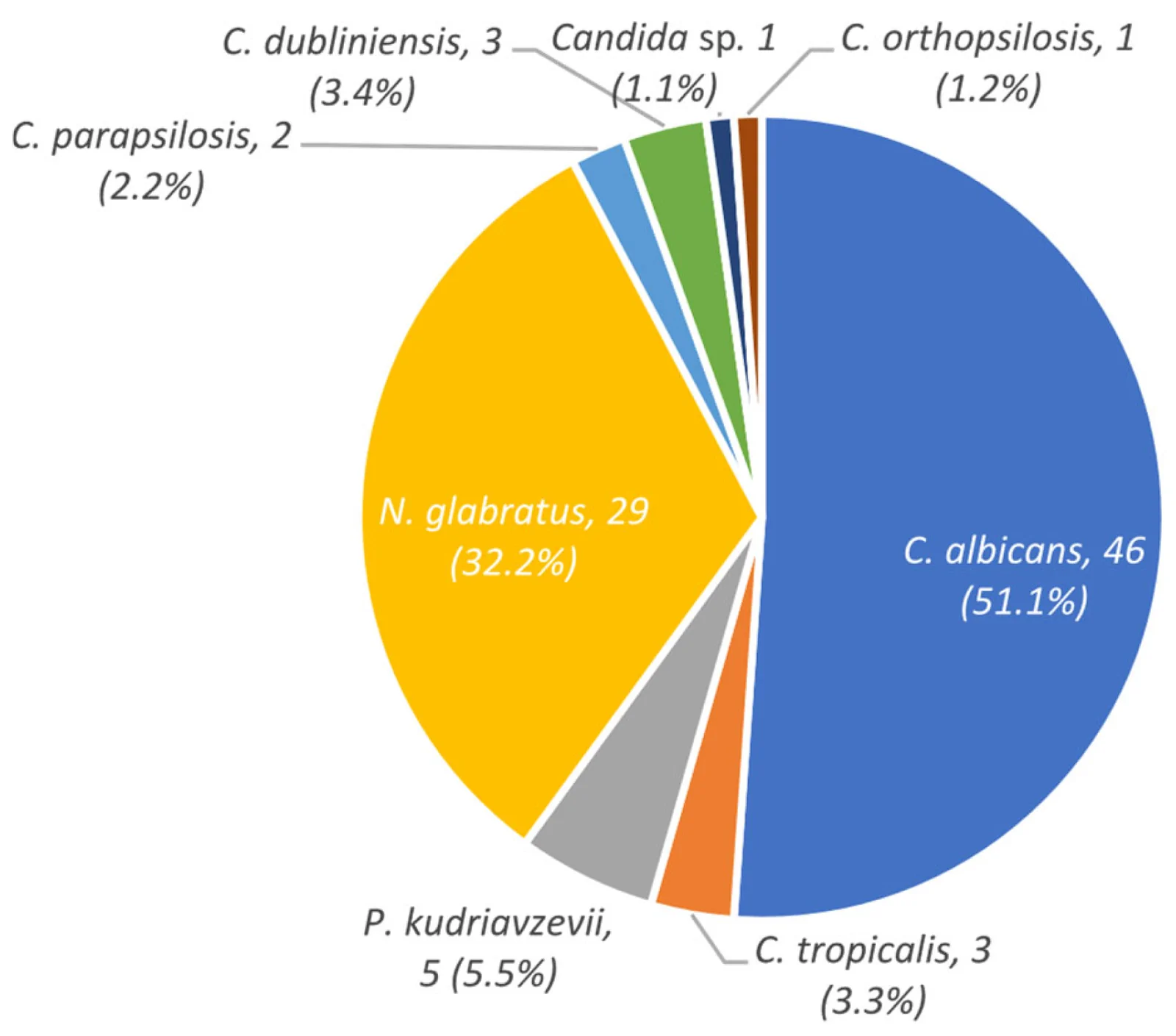
Distribution of *Candida* species identified in clinical samples from patients. This distribution reflects a clinical profile characterized by the predominance of *C. albicans* (51.1%), along with a considerable proportion of non-*albicans Candida* species (48.9%). Thirty-three isolates were molecularly confirmed (see Appendix A.1).

On the other hand, the results of *Candida* species identification obtained using the BD Phoenix™ automated system, CHROMagar™ Candida, physiological tests, PCR, and ITS region sequencing were compared (Table 4 and Table A1). In total, seven yeast species were identified from ninety clinical samples corresponding to eighty-eight patients. Figure 3 shows the distribution of the *Candida* species identified, as well as the characterization methods used (see Table 4). Of 33 isolates compared using culture methods, 20 matched the molecular identification (PCR and phylogenetic analysis). In addition, 19 matches and 14 discrepancies were observed between the BD Phoenix automated system and molecular tests (see Table A1).

In addition, two mixed cultures were detected, in which *N. glabratus* and *P. kudriavzevii* coexisted. The most frequently identified species was *C. albicans* (46/90; 51.1%), followed by *N. glabratus* (29/90; 32.2%). Less frequent species included *P. kudriavzevii* (5/90; 5.5%), *C. tropicalis* (3/90; 3.3%), *C. dubliniensis* (3/90; 3.3%), *C. parapsilosis* (2/90; 2.2%), *C. orthopsilosis* (1/90; 1.1%), and *Candida* sp. (1/90; 1.1%).

## 4. Discussion

The findings of this study highlight the predominance of *Candida* infection/colonization in older adults, especially patients aged 60 years or older, which is consistent with previous reports emphasizing that, as age advances, the immune system undergoes progressive deterioration resulting in increased vulnerability to infections; reduced immunization efficacy; and a higher incidence of conditions such as cancer, autoimmune diseases, and chronic illnesses [35,36,37,38]. Among the most common factors associated with nosocomial candidiasis are increased exposure to invasive procedures, prolonged hospitalizations, the use of medical devices, and intrinsic factors such as components of innate and adaptive immunity that are altered by biological aging [35]. The literature indicates that various forms of candidiasis, including candiduria, esophageal infection, and oral infection, are particularly common in geriatric patients with comorbidities such as diabetes mellitus, prolonged use of antibiotics, presence of permanent urinary catheters, and malignant diseases. Additional factors such as corticosteroid use, obstructive pulmonary disease, acid suppression, esophageal dysmotility, and HIV/AIDS also contribute to susceptibility in this population [36,37,38]. On the other hand, this study observed a higher prevalence of *C. albicans*, followed by *N. glabratus*, which coincides with various microbiological studies that have identified species such as *C. albicans*, *N. glabratus*, *C. parapsilosis*, and *C. tropicalis* in the oral cavity, with *N. glabratus* being more frequent in older adults [39,40].

The distribution of *Candida* by sex reveals a higher prevalence in women (60.2%), which could be linked to physiological and hormonal differences influencing microbiota and immune responses [41]. This trend coincides with other epidemiological studies, such as the one described by Torres Guerrero et al. (2014), which investigated *Candida* spp. infections in a secondary-level hospital and observed that women accounted for 65.49% of reported mycoses [13].

The predominant distribution of *C. albicans* (51.1%; 46/90), especially in urinary and respiratory samples, along with a significant proportion (46.7%; 42/90) of non-*albicans* species, mainly *N. glabratus* (32.2%; 29/90), aligns with findings from other tertiary hospitals in Mexico, such as the Regional High Specialty Hospital of Ixtapaluca [42] and the Juárez Hospital of Mexico [14].

*C. albicans* is the main etiological agent of vulvovaginal candidiasis (VVC), responsible for approximately 85–90% of cases. Various studies have reported a higher VVC prevalence in pregnant women than in non-pregnant women, which is associated with factors such as hormonal changes, since increased concentrations of sex hormones like estrogen during pregnancy create a carbon-rich environment (increased vaginal glycogen), and other factors like diabetes mellitus and hygiene conditions [41,43]. In this study, VVC accounted for 14.7% (n = 13/88) of cases, primarily caused by *N. glabratus*, followed by *C. albicans*. This finding differs from that of previous studies in different hospitals in Mexico, which report *C. albicans* as the most common species in vaginal infections, followed by *P. kudriavzevii* [44]. Epidemiological studies worldwide identify *N. glabratus* as the second most prevalent species in this infection type [45]. Our patients presented underlying clinical diagnoses considered determining factors in this clinical manifestation, including diabetes/ketoacidosis and pregnancy. This aligns with various reports that include these predisposing factors, as well as hormonal imbalance, poorly controlled diabetes, immunosuppression, broad-spectrum antibiotic use, glucocorticoids, and genetic predispositions [44].

In patients with *Candida* isolates from the urinary tract, *C. albicans* (22/90) was the most frequently recovered species, followed by *N. glabratus* (14/90). Women were the most affected (27/88). These findings are consistent with those of other studies that have reported that more than 50% of *Candida* species isolated from urine samples correspond to *C. albicans*, followed by *N. glabratus*, *C. tropicalis*, and *C. parapsilosis* [46,47]. *Candida* yeasts are the main etiological agents of urinary tract infections; however, distinguishing between colonization, contamination, and infection is a clinical challenge due to the lack of standardized diagnostic criteria [47,48]. According to the literature, the most common risk factors for urinary tract infections include advanced age, female sex, and antibiotic use [47]. Diagnostic studies, such as urinary tract imaging, blood cultures, cultures from other non-sterile areas, and the assessment of risk factors, are essential for identifying invasive candidiasis [48].

Respiratory tract involvement was the second most frequent manifestation (19.3%; 17/88), with *C. albicans* being the most common etiological agent, followed by *N. glabratus*. This finding is relevant in patients with respiratory and systemic comorbidities, as well as in those with a history of hospitalization or exposure to invasive devices. The most affected age group comprised older adults (≥60 years), which is consistent with epidemiological data linking advanced age; immunological alterations; and greater exposure to predisposing comorbidities such as chronic obstructive pulmonary disease (COPD), type 2 diabetes mellitus, and chronic kidney disease [35,36,37]. The history of SARS-CoV-2 infection in two patients indicates an increased risk of opportunistic fungal infections, as various authors have mentioned that COVID-19 infection can promote the invasion of commensal yeasts and contribute to the development of invasive fungal infections. Patients with severe COVID-19 are more susceptible to healthcare-associated infections (HAIs), including candidemia, especially those who receive corticosteroids and broad-spectrum antibiotics or who are immunosuppressed and nutritionally deficient [49,50]. The identification of *Candida* species in the respiratory tract through clinical laboratory studies is mainly based on their recovery from cultures. However, neither culture-based tests nor molecular techniques applied to respiratory samples allow for a definitive distinction between contamination, colonization, or invasive infection. Direct microscopic examination, used in samples such as bronchoalveolar lavage, offers greater sensitivity than culture, but its application is limited by the need for expertise in interpretation and the difficulty in distinguishing between fungi with similar morphology [51]. Although the detection of yeast in respiratory secretions is usually considered indicative of colonization, in immunosuppressed patients, those undergoing mechanical ventilation, or those with chronic lung disease, this condition can progress to invasive pulmonary candidiasis [5,52].

The incidence of invasive candidiasis observed in our study population (10.2%; n = 9/88) is consistent with that in previous reports highlighting an increase in opportunistic fungal infections in patients with comorbidities or invasive medical devices [53,54]. The predominance of *C. albicans* as the etiological agent found in this study remains consistent with its recognition as the most common species involved in invasive infections [11,54]. The recovery of *P. kudriavzevii* and *C. parapsilosis*, the former from a patient with candidemia associated with chronic kidney disease and the latter from a patient with peritonitis associated with peritoneal dialysis, is clinically relevant, since the presence of *C. parapsilosis* has been associated with the use of intravascular devices and its ability to form biofilms [47]. The predominant distribution between the ages of 29 and 39 suggests a significant impact on young patients with acute clinical conditions, such as pancreatitis and peritonitis associated with dialysis, both previously described as predisposing factors in *Candida* infections [11,48]. On the other hand, diagnoses such as Ludwig’s angina with soft tissue sepsis with a history of surgical wounds and Fournier’s gangrenous wounds are indicative of fungal colonization of altered anatomical environments with a high microbial load, where *Candida* can change from a commensal to an invasive pathogen [11].

Cutaneous candidiasis accounted for 4.5% (n = 4/88) of documented cases, predominantly in male patients. The clinical history recorded, including nephrostomy, surgical dehiscence, and Penrose drain secretion, coincides with the risk factors described in the literature, commonly associated with colonization or infection of moist areas compromised by inflammatory or surgical processes or the presence of medical devices [1].

The results obtained in this study showed discrepancies between conventional identification methods (biochemical and physiological methods). Of 33 isolates compared using culture methods, 21 matched the molecular identification (PCR and phylogenetic analysis). This finding is consistent with what has been reported in the literature, demonstrating insufficient differentiation between closely related species or those with atypical metabolic profiles, which can compromise diagnostic accuracy [55,56]. On the other hand, 20 matches and 13 discrepancies were observed between the BD Phoenix automated system and molecular tests, reflecting limitations in identification that have been documented in the literature. Marucco et al. [57] compared BD Phoenix with MALDI-TOF MS and pointed out that, although the automated system offers speed and clinical coverage, it can fail to identify less common or phylogenetically close species. Kanesaka et al. [58] also highlighted that BD Phoenix has difficulties in differentiating yeasts with atypical biochemical profiles. Given this scenario, molecular techniques have become more relevant, as they allow for the rapid and accurate identification and characterization of various *Candida* species, even those with similar phenotypic profiles [56]. The discrepancy between phenotypic and molecular methods means that results regarding species distribution should be interpreted with caution. Conventional methods tend to oversimplify or distort diversity, while molecular techniques provide a more accurate and reliable view of the actual composition of *Candida* species distribution. More than one-third of phenotypic identifications were inconsistent, reducing the reliability of the results based solely on biochemical or phenotypic tests. Therefore, any analysis of species distribution should be interpreted with caution. Evidence suggests that only identifications confirmed by molecular techniques provide a solid basis for characterizing *Candida* epidemiology.

Generally, non-*albicans Candida* species are associated with greater resistance and high mortality rates [59]. Recently, there have been more frequent reports of studies indicating intrinsic and acquired resistance to multiple drugs (azoles, echinocandins, and polyenes), which has been most notable in *N. glabratus* [60]. *N. glabratus* infections represent a growing challenge due to their increased incidence and reduced sensitivity to conventional antifungals, and they have become a cause of candidemia with high mortality rates in many regions of the world. Its resistance to azoles complicates treatment, and although echinocandins are the therapy of choice for invasive candidiasis, their limited availability in low- and middle-income countries forces the continued use of less effective azoles [61].

The presence of species such as *N. glabratus*, *P. kudriavzevii*, *C. dubliniensis*, *C. parapsilosis*, and *C. orthopsilosis* is clinically relevant, given their resistance profiles reported in the literature. Studies conducted in the US have reported higher rates of resistance to fluconazole: 4 to 9%, 2 to 6%, and 11 to 13% for *C. tropicalis*, *C. parapsilosis*, and *N. glabratus*, respectively [62,63]. It should be emphasized that antifungal susceptibility testing was not performed in the present study; the resistance profiles mentioned above correspond exclusively to data reported in the literature and do not reflect the susceptibility of the isolates recovered in this work.

Among the limitations of this study are the following: it was not possible to obtain data such as definitive diagnosis, patient progression, or antifungal treatment administered, which limits the ability to establish relationships between variables. Furthermore, with regard to medical history, some records were incomplete or contained inconsistencies in the data, which reduced the effective size of the analyzable sample. Because molecular identification tests were only performed on 33 isolates that presented discrepancies between physiological and biochemical methods, phylogenetic characterization was restricted to only a fraction of the cases. Several additional limitations should be acknowledged. First, the single-center, retrospective design and the relatively small sample size, particularly for rare species and invasive disease, limit the generalizability of the findings to other institutions or regions. Second, because molecular confirmation was applied selectively to discrepant isolates rather than to the entire collection, the proportion of phenotypic identifications that were independently verified is limited (selection bias), and the accuracy of phenotypic methods may be overestimated for the non-discrepant isolates (verification bias). Third, antifungal susceptibility testing was not performed, so resistance patterns could not be assessed. Fourth, identification did not include MALDI-TOF mass spectrometry, currently considered a reference method in many clinical laboratories, or specific screening for Candida auris. Finally, the study period (2021–2022) overlapped with the COVID-19 pandemic, a context that may have influenced the observed species distribution.

Among the recommendations for future research, it is suggested to increase the number of clinical isolates and hospitals involved to improve the representativeness and generalization of the results. Molecular identification techniques should be implemented in all isolates, not only in discrepant ones, to improve diagnostic accuracy.

## 5. Conclusions

Our findings showed that the majority of infected patients were women (60.2%) and older adults (≥60 years). Candida was most frequently recovered from the urinary tract (47.7%), followed by the respiratory tract (19.3%), vulvovaginal tract (14.7%), invasive infection (10.2%), and skin infection (4.5%). Although *C. albicans* was the predominant species (51.1%), the significant presence of species belonging to the non-*albicans* family (n = 44/90; 48.9%) in specific clinical contexts highlights the urgency of monitoring the epidemiological profile. The diversity observed in this study may be due to the influence of institutional factors (such as the diagnostic methods used), clinical factors (such as immune status, the use of invasive devices, or comorbidities), and microbiological aspects in the identification of these opportunistic yeasts.

This research identified the diversity of *Candida* species isolated from patients with different clinical histories at a tertiary care hospital in Puebla. Phylogenetic analysis allowed for more precise identification of isolates with discrepancies, highlighting the usefulness of complementing conventional methods with molecular methods. However, the lack of agreement between phenotypic and molecular methods suggests that the results regarding species distribution should be interpreted with caution. Furthermore, it is important to mention that the implementation of these techniques can be affected by the high cost of supplies, laboratory materials, and trained personnel, hindering access to accurate and timely diagnoses. For clinical practice, these findings support strengthening mycological surveillance in tertiary care settings and incorporating molecular confirmation, or other reference methods such as MALDI-TOF mass spectrometry, into routine identification algorithms when phenotypic results are ambiguous. Future studies should be multicentric, include antifungal susceptibility testing and clinical outcome data, and integrate the WHO fungal priority pathogens framework to better inform local antifungal stewardship.

## Figures and Tables

**Table 1:** Identification of *Candida* species based on amplicon size. Adapted from García-Salazar et al. [22].

Species	Amplicon Size (pb)
*C. albicans*	850
*N. glabratus*	1000
*C. tropicalis*	790
*C. parapsilosis*	731
*P. kudriavzevii*	800
*M. guilliermondii*	1100
*C. lusitaniae*	590
*C. dubliniensis*	810

**Table 2:** Reference sequences retrieved from GenBank and included in the phylogenetic analysis.

Species/Reference Strain	GenBank Accession Numbers
*Candida parapsilosis*	LC389747.1; EU564206.1; EU552502.1; LC389733.1; KY102294.1; KM014590.1; EU564200.1; EU552499.1; EU564205.1; MF767638.1
*Candida metapsilosis*	EU484055.1; KM014585.1
*Candida orthopsilosis*	KJ451699.1; EU552495.1; EU557371.1; KY102262.1; KC846140.1; KF738147.1; EU557370.1
*Candida tropicalis*	KY102470.1; OR859805.1; MH793862.1; MH545915.1
*Candida albicans*	KF241849.1; KC905077.1; KF241847.1
*Candida dubliniensis*	KP131696.1; KP131697.1; KC905080.1
*Clavispora lusitaniae*	KY102563.1; AY493434.1; KP131851.1
*Candida haemulonis*	JX459674.1; JX459675.1
*Candida auris*	KT305985.1; KT305984.1
*Meyerozyma guilliermondii*	MH545918.1
*Debaryomyces hansenii* var. *hansenii*	GQ376085.1
*Debaryomyces hansenii*	PQ143187.1
*Diutina rugosa*	GU144663.1; KT336717.1
*Yarrowia lipolytica*	KP132909.1; KP132908.1
*Kluyveromyces marxianus*	KC905771.1; HQ396523.1
*Pichia norvegensis*	AB278166.1; AB278165.1
*Nakaseomyces bracarensis*	JN882338.1; JN882340.1; KP674833.1
*Nakaseomyces nivariensis*	JN183443.1; PV053621.1; OR690735.1; OR690737.1; KP068747.1; KP068746.1; KP131740.1
*Nakaseomyces glabratus*	KC408956.1; MF187263.1; MF187253.1; KP675693.1; MF371417.1; KY575045.1; KC408962.1; KP674942.1; KP675655.1; LC317496.1; KP675517.1; OW988753.1; ON479770.1; KP068740.1
*Pichia kudriavzevii*	KC886644.1; MK394162.1; KP674762.1; MK592834.1; MZ677198.1; FJ231424.1

**Table 3:** Comparison of identification methods applied to 33 yeast isolates. Results obtained with BD Phoenix, CHROMagar Candida, phenotypic tests (chlamydoconidia, germ tube, thermotolerance), and PCR are included. PCR Cand directly identified 22 isolates (highlighted in blue); 15 were characterized as *C. albicans* and 7 as *N. glabratus*. Additionally, 11 isolates that showed discrepancies between previous tests were characterized by sequencing of the ITS region.

N° ID	BD Phoenix	CHROMagar	Phenotypic Tests			PCR (pb)	PCR + ITS Phylogenetic Analysis
			Chlamydoconidia	Germ Tube	Thermotolerance		
**6**	*C. albicans*	*C. albicans*	−	+	+	*C. albicans* (850)	*
**13**	*C. albicans*	*C. albicans*	−	+	+	*C. albicans* (850)	*
**19**	*C. albicans*	*C. albicans*	−	+	+	*C. albicans* (850)	*
**20**	*C. albicans*	*C. albicans*	−	+	+	*C. albicans* (850)	*
**21**	*C. albicans*	*C. albicans*	−	+	+	*C. albicans* (850)	*
**31**	*N. glabratus*	*P. kudriavzevii*				*N. glabratus* (1000)	*
**33**	*N. glabratus*	*P. kudriavzevii*				*N. glabratus* (1000)	*
**37**	*C. dubliniensis*	*N. glabratus*				*N. glabratus* (1000)	*
**40**	*P. kudriavzevii*	*N. glabratus*				*C. albicans* (850)	*P. kudriavzevii*
**41**	*C. melibiosica*	*P. kudriavzevii*				*N. glabratus* (1000)	*N. glabratus*
**42**	*C. tropicalis*	*C. albicans*	+	+	+	*C. albicans* (850)	*
**43**	*C. kefyr*	*P. kudriavzevii*				*N. glabratus* (1000)	*N. glabratus*
**48**	*C. albicans*	*C. albicans*	−	+	+	*C. albicans* (850)	*
**49**	*C. albicans*	*C. albicans*	+	−	−	*C. albicans* (850)	*
**53**	*C. albicans*	*N. glabratus*	−	+	+	*C. albicans* (850)	*
**55**	*N. glabratus*	*P. kudriavzevii*				*C. albicans* (850)	*P. kudriavzevii*
**1b**	*C. parapsilosis*	*Candida* sp.				*C. albicans* (850)	*C. parapsilosis*
**4b**	*C. albicans*	*Candida* sp.	+	−	+	*C. albicans* (850)	*
**6b**	*C. albicans*	*N. glabratus*	+	−	+	*N. glabratus* (1000)	*
**7b**	*C. albicans*	*P. kudriavzevii*	−	−	+	*N. glabratus* (1000)	*C. parapsilosis*
**8b**	*C. dubliniensis*	*C. albicans*	+	+	+	*C. albicans* (850)	*
**13b**	*P. kudriavzevii*	*N. glabratus*				*C. albicans* (850)	*P. kudriavzevii*
**17b**	*C. tropicalis*	*C. tropicalis*				*C. albicans* (850)	*C. tropicalis*
**18b**	*C. albicans*	*N. glabratus*	−	−	+	*N. glabratus* (1000)	*N. glabratus*
**20b**	*C. albicans*	*C. albicans*	−	+	+	*C. albicans* (850)	*
**22b**	*C. albicans*	*C. albicans*	−	+	+	*C. albicans* (850)	*
**24b**	*C. albicans*	*C. albicans*	−	+	+	*C. albicans* (850)	*
**25b**	*C. parapsilosis*	*Candida* sp.				*C. albicans* (850)	*N. glabratus*
**31b**	*N. glabratus*	*P. kudriavzevii*				*N. glabratus* (1000)	*
**35b**	*C. albicans*	*N. glabratus*	−	−	+	*N. glabratus* (1000)	*
**36b**	*C. tropicalis*	*N. glabratus*				*N. glabratus* (1000)	*
**44b**	*C. parapsilosis*	*Candida* sp.				*C. albicans* (850)	*C. orthopsilosis*
**47b**	*C. tropicalis*	*C. tropicalis*				*C. albicans* (850)	*

+ = Positive for the physiological test. − = Negative for the physiological test. * Non discrepancies.

**Table 4:** Identification of clinical *Candida* isolates using various phenotypic and molecular methods. The columns are not additive: the “Biochemical/Physiological” column shows the initial phenotypic count, whereas the “PCR” and “PCR + ITS Phylogenetic Analysis” columns indicate the molecular results obtained for the 33 discrepant isolates. The “Final Identification” column reports the net number of isolates per species after reconciling all methods, including reassignments of isolates initially misidentified by phenotypic methods.

Identified Species	Biochemical/Physiological (n)	PCR (n)	PCR + ITS Phylogenetic Analysis (n)	Final Identification	Observations
***C. albicans***	48	15	0	46 (*C. albicans*)	Confirmed by physiological tests; some isolates corrected by PCR
***N. glabratus***	22	7	4	29 (*N. glabratus*)	Three isolates corrected by ITS (misidentified as *C. kefyr*, *C. melibiosica*, and *C. albicans*)
***C. tropicalis***	5	0	1	3 (*C. tropicalis*)	One isolate confirmed by phylogenetic analysis
***P. kudriavzevii***	4	0	3	5 (*P. kudriavzevii*)	Three isolates corrected by ITS
***C. parapsilosis***	3	0	2	2 (*C. parapsilosis*)	Molecular confirmation by ITS
***C. dubliniensis***	5	0	0	3 (*C. dubliniensis*)	Identified by physiological tests (one isolate reassigned as *N. glabratus* by PCR)
***C. kefyr***	1	0	0	0 (*C. kefyr*)	Reassigned as *N. glabratus* by ITS analysis
***C. melibiosica***	1	0	0	0 (*C. melibiosica*)	Reassigned as *N. glabratus* by ITS analysis
***C. orthopsilosis***	0	0	1	1 (*C. orthopsilosis*)	Identified exclusively by phylogenetic analysis
***Candida* sp.**	1	0	0	1 (*Candida* sp.)	Isolate not recovered
***N***	90	22	11	90	

## Data Availability

Data are available on request.

## Supplementary Materials

The following supporting information can be downloaded at: https://www.mdpi.com/article/10.3390/epidemiologia7040112/s1, Table S1: Isolates and accession numbers from Genbank of the sequences obtained with the ITS marker; Figure S1: The phylogenetic tree was constructed from ITS sequences using the maximum likelihood method using MEGAX Bootstrap support values are displayed at nodes.

## Appendix A

### Appendix A.1

**Table A1:** Comparison of microbiological identification methods in eighty-eight clinical isolates of the genus *Candida*. Results obtained using BD Phoenix™, CHROMagar™ *Candida*, physiological tests (chlamydoconidia, germ tube test, and thermotolerance), PCR, and phylogenetic analysis are included. The “FINAL ID” column corresponds to the definitive identification based on the integration of all methods.

N° de ID	BD Phoenix	CHROMagar	Chlamydoconidia	Germ Tube	Thermotolerance	PCR Cand (pb)	PCR + ITS Phylogenetic Analysis	Final ID
1	*C. albicans*	*C. albicans*	Positive	Positive	Positive			*C. albicans*
2	*C. albicans*	*C. albicans*	Positive	Positive	Positive			*C. albicans*
6	*C. albicans*	*C. albicans*	Negative	Positive	Positive	*C. albicans* (850)		*C. albicans*
7	*C. albicans*	*C. albicans*	Positive	Positive	Positive			*C. albicans*
8	*C. albicans*	*C. dubliniensis*	Negative	Negative	Negative			*C. dubliniensis*
9	*C. albicans*	*C. dubliniensis*	Negative	Negative	Negative			*C. dubliniensis*
10	*C. albicans*	*C. albicans*	Positive	Positive	Positive			*C. albicans*
11	*C. albicans*	*C. albicans*	Positive	Positive	Positive			*C. albicans*
12	*C. albicans*	*C. tropicalis*	Positive	Positive	Positive			*C. albicans*
13	*C. albicans*	*C. albicans*	Negative	Positive	Positive	*C. albicans* (850)		*C. albicans*
15	*C. albicans*	*C. albicans*	Positive	Positive	Positive			*C. albicans*
18	*C. albicans*	*C. albicans*	Positive	Positive	Positive			*C. albicans*
19	*C. albicans*	*C. albicans*	Negative	Positive	Positive	*C. albicans* (850)		*C. albicans*
20	*C. albicans*	*C. albicans*	Negative	Positive	Positive	*C. albicans* (850)		*C. albicans*
21	*C. albicans*	*C. albicans*	Negative	Positive	Positive	*C. albicans* (850)		*C. albicans*
26	*N. glabratus*	*N. glabratus*						*N. glabratus*
28	*N. glabratus*	*N. glabratus*						*N. glabratus*
29	*N. glabratus*	*N. glabratus*						*N. glabratus*
30	*N. glabratus*	*N. glabratus*						*N. glabratus*
31	*N. glabratus*	*P. kudriavzevii*				*N. glabratus* (1000)		*N. glabratus*
33	*N. glabratus*	*P. kudriavzevii*				*N. glabratus* (1000)		*N. glabratus*
34	*N. glabratus*	*P. kudriavzevii*						*N. glabratus*
35	*N. glabratus*	*P. kudriavzevii*						*N. glabratus*
36	*N. glabratus*	*P. kudriavzevii*						*N. glabratus*
37	*C. dubliniensis*	*N. glabratus*				*N. glabratus* (1000)		*N. glabratus*
38	*C. dubliniensis*	*C. tropicalis*						*C. dubliniensis*
39	*C. dubliniensis*	*C. albicans*	Positive	Positive	Positive			*C. albicans*
40	*P. kudriavzevii*	*N. glabratus*				*C. albicans* (850)	*P. kudriavzevii*	*P. kudriavzevii*
41	*C. melibiosica*	*P. kudriavzevii*				*N. glabratus* (1000)	*N. glabratus*	*N. glabratus*
42	*C. tropicalis*	*C. albicans*	Positive	Positive	Positive	*C. albicans* (850)		*C. albicans*
43	*C. kefyr*	*P. kudriavzevii*				*N. glabratus* (1000)	*N. glabratus*	*N. glabratus*
45	*C. albicans*	*C. tropicalis*	Positive	Positive	Positive			*C. albicans*
46	*C. albicans*	*C. tropicalis*	Positive	Positive	Positive			*C. albicans*
47	*C. albicans*	*C. tropicalis*	Positive	Positive	Positive			*C. albicans*
48	*C. albicans*	*C. albicans*	Negative	Positive	Positive	*C. albicans* (850)		*C. albicans*
49	*C. albicans*	*C. albicans*	Positive	Negative	Negative	*C. albicans* (850)		*C. albicans*
50	*C. albicans*	*C. tropicalis*	Positive	Positive	Positive			*C. albicans*
51	*C. albicans*	*C. tropicalis*	Positive	Positive	Positive			*C. albicans*
52	*C. albicans*	*C. tropicalis*	Positive	Positive	Positive			*C. albicans*
53	*C. albicans*	*N. glabratus*	Negative	Positive	Positive	*C. albicans* (850)		*C. albicans*
54	*C. albicans*	*C. albicans*	Positive	Positive	Positive			*C. albicans*
55	*N. glabratus*	*P. kudriavzevii*				*C. albicans* (850)	*P. kudriavzevii*	*P. kudriavzevii*
56	*N. glabratus*	*N. glabratus*						*N. glabratus*
1b	*C. parapsilosis*	*Candida* sp.				*C. albicans* (850)	*C. parapsilosis*	*C. parapsilosis*
2b	*N. glabratus*	*N. glabratus*						*N. glabratus*
3b	*P. kudriavzevii*	*P. kudriavzevii*						*P. kudriavzevii*
4b	*C. albicans*	*Candida* sp.	Positive	Negative	Positive	*C. albicans* (850)		*C. albicans*
5b	*N. glabratus*	*N. glabratus*						*N. glabratus*
6b	*C. albicans*	*N. glabratus*	Positive	Negative	Positive	*N. glabratus* (1000)		*N. glabratus*
7b	*C. albicans*	*P. kudriavzevii*	Negative	Negative	Positive	*N. glabratus* (1000)	*C. parapsilosis*	*C. parapsilosis*
8b	*C. dubliniensis*	*C. albicans*	Positive	Positive	Positive	*C. albicans* (850)		*C. albicans*
9b	*C. dubliniensis*	*C. albicans*	Positive	Positive	Positive			*C. albicans*
10b	*N. glabratus*	*N. glabratus*						*N. glabratus*
11b	*P. kudriavzevii*	*P. kudriavzevii*						*P. kudriavzevii*
12b	*C. albicans*	*C. albicans*	Positive	Positive	Positive			*C. albicans*
13b	*P. kudriavzevii*	*N. glabratus*				*C. albicans* (850)	*P. kudriavzevii*	*P. kudriavzevii*
14b	*N. glabratus*	*N. glabratus*						*N. glabratus*
15b	*C. albicans*	*C. albicans*	Positive	Positive	Positive			*C. albicans*
16b	*C. albicans*	*C. albicans*	Negative	Positive	Positive			*C. albicans*
17b	*C. tropicalis*	*C. tropicalis*				*C. albicans* (850)	*C. tropicalis*	*C. tropicalis*
18b	*C. albicans*	*N. glabratus*	Negative	Negative	Positive	*N. glabratus* (1000)	*N. glabratus*	*N. glabratus*
19b	*C. dubliniensis*	*C. albicans*	Positive	Positive	Positive			*C. albicans*
20b	*C. albicans*	*C. albicans*	Negative	Positive	Positive	*C. albicans* (850)		*C. albicans*
21b	*C. albicans*	*C. albicans*	Positive	Positive	Positive			*C. albicans*
22b	*C. albicans*	*C. albicans*	Negative	Positive	Positive	*C. albicans* (850)		*C. albicans*
23b	*C. albicans*	*C. albicans*	Positive	Positive	Positive			*C. albicans*
24b	*C. albicans*	*C. albicans*	Negative	Positive	Positive	*C. albicans* (850)		*C. albicans*
25b	*C. parapsilosis*	*Candida* sp.				*C. albicans* (850)	*C. parapsilosis*	*C. parapsilosis*
26b	*C. tropicalis*	*C. tropicalis*						*C. tropicalis*
27b	*N. glabratus*	*N. glabratus*						*N. glabratus*
28b	*C. albicans*	*C. albicans*	Positive	Positive	Positive			*C. albicans*
29b	*C. tropicalis*	*C. tropicalis*						*C. tropicalis*
30b	*Candida* sp.	*Candida* sp.						*Candida* sp.
31b	*N. glabratus*	*P. kudriavzevii*				*N. glabratus* (1000)		*N. glabratus*
32b	*N. glabratus*	*N. glabratus*						*N. glabratus*
33b	*N. glabratus*	*N. glabratus*						*N. glabratus*
34b	*C. albicans*	*C. albicans*	Positive	Positive	Positive			*C. albicans*
35b	*C. albicans*	*N. glabratus*	Negative	Negative	Positive	*N. glabratus* (1000)		*N. glabratus*
36b	*C. tropicalis*	*N. glabratus*				*N. glabratus* (1000)		*N. glabratus*
37b	*C. albicans*	*C. albicans*	Positive	Positive	Positive			*C. albicans*
38b	*C. albicans*	*C. albicans*	Positive	Positive	Positive			*C. albicans*
39b	*C. albicans*	*C. albicans*	Positive	Positive	Positive			*C. albicans*
40b	*N. glabratus*	*N. glabratus*						*N. glabratus*
41b	*N. glabratus*	*N. glabratus*						*N. glabratus*
42b	*C. albicans*	*C. albicans*	Positive	Positive	Positive			*C. albicans*
43b	*C. dubliniensis*	*C. albicans*	Positive	Positive	Positive			*C. albicans*
44b	*C. parapsilosis*	*Candida* sp.				*C. albicans* (850)	*C. orthopsilosis*	*C. orthopsilosis*
45b	*N. glabratus*	*N. glabratus*						*N. glabratus*
46b	*C. albicans*	*C. albicans*	Positive	Positive	Positive			*C. albicans*
47b	*C. tropicalis*	*C. tropicalis*				*C. albicans* (850)		*C. albicans*

### Appendix A.2

**Table A2:** Distribution of patients by age group and type of colonization/infection, differentiated by sex (F: female; M: male). Values are expressed as number of cases (N) and relative percentage (%).

Variables	Sex		
	F	M	N (%)
	n = 53	n = 35	N = 88
**Age**	42.7 ± 19.9	51.88 ± 16.1	46.39 ± 18.9
13–19	6	2	8 (9.09)
19–29	9	0	9 (10.23)
29–39	10	3	13 (14.77)
39–49	7	10	17 (19.32)
49–59	10	9	19 (21.59)
≥60	11	11	22 (25.00)
**Types of colonization/infection**			
Balanitis	-	1	1 (1.13)
Candidemia	-	2	2 (2.27)
Cutaneous candidiasis	1	3	4 (4.54)
Invasive candidiasis	5	4	9 (10.22)
Vulvovaginal tract	13	-	13 (14.77)
Respiratory tract colonization	7	10	17 (19.31)
Urinary tract colonization	27	15	42 (47.72)
