# Supplementary material for: Using Phenotypic, Biochemical, and Molecular Methods to Identify Candida Species Isolated in a Tertiary Hospital in Mexico
Source: Epidemiologia (Basel). 2026 Aug 17;7(4):112. doi: 10.3390/epidemiologia7040112 (PMC13512195; doi:10.3390/epidemiologia7040112)
Supplement: Supplementary file 1 [file epidemiologia-07-00112-s001.zip › epidemiologia-4344105 supplementary figure S1.pdf]

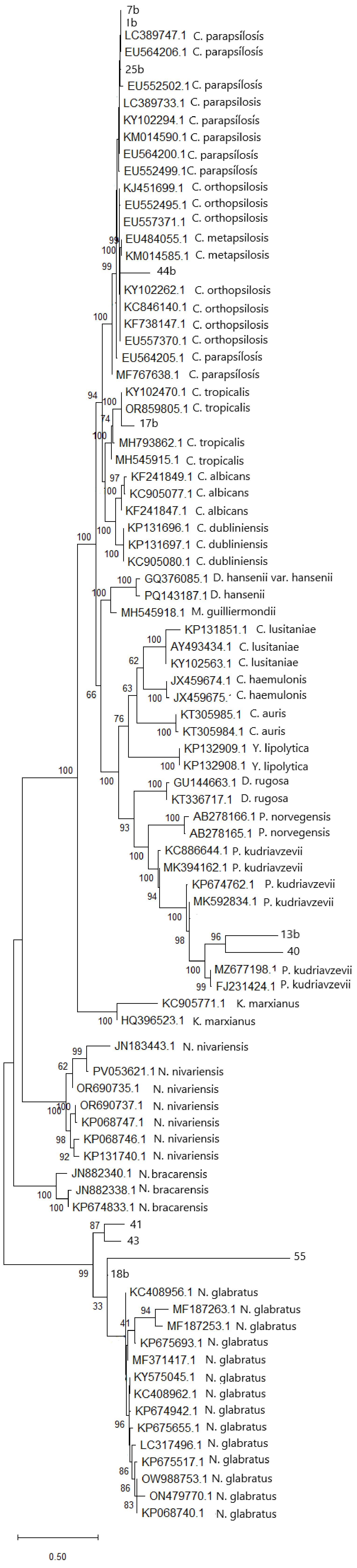

Figure S1. The phylogenetic tree was constructed from ITS sequences using the maximum likelihood method using MEGAX. Bootstrap support values are displayed at nodes.
