# Supplementary material for: Using Phenotypic, Biochemical, and Molecular Methods to Identify Candida Species Isolated in a Tertiary Hospital in Mexico
Source: Epidemiologia (Basel). 2026 Aug 17;7(4):112. doi: 10.3390/epidemiologia7040112 (PMC13512195; doi:10.3390/epidemiologia7040112)
Supplement: Supplementary file 1 [file epidemiologia-07-00112-s001.zip › epidemiologia-4344105 Supplementary Table S1.pdf]

Supplementary Table S1. Isolates and accession numbers from Genbank of the sequences obtained with the ITS marker.

**Isolate**	**Accession Number**
1b	PZ325472
7b	PZ325473
25b	PZ325474
44b	PZ325475
13b	PZ325476
17b	PZ325477
18b	PZ325478
40	PZ325479
41	PZ325480
43	PZ325481
55	PZ325482
